# Defining Transformative Experiences: A Conceptual Analysis

**DOI:** 10.3389/fpsyg.2022.790300

**Published:** 2022-06-24

**Authors:** Alice Chirico, Marta Pizzolante, Alexandra Kitson, Elena Gianotti, Bernhard E. Riecke, Andrea Gaggioli

**Affiliations:** ^1^Department of Psychology, Research Center in Communication Psychology, Universitá Cattolica del Sacro Cuore, Milan, Italy; ^2^School of Interactive Arts and Technology (SIAT), Simon Fraser University, Surrey, BC, Canada; ^3^Department of Psychology, Universitá Cattolica del Sacro Cuore, Milan, Italy; ^4^Applied Technology for Neuro-Psychology Lab, I.R.C.C.S. Istituto Auxologico Italiano, Milan, Italy

**Keywords:** transformative experiences, psychological change, conceptual analysis, complex emotions, transcendence

## Abstract

The concept of transformative experience (TE) has been widely explored by several disciplines from philosophy to neurobiology, and in different domains, from the spiritual to the educational one. This attitude has engendered heterogeneous models to explain this phenomenon. However, a consistent and clear understanding of this construct remains elusive. The aim of this work is to provide an initial comprehensive interdisciplinary, cross-domain, up-to-date, and integrated overview on the concept of TEs. Firstly, all the models and theories on TEs were reviewed to extract and analyze TEs’ main components emerging from different disciplines. Then, this preliminary analysis was integrated with an in-depth examination of redundancies and particularities across domains and disciplines, to provide an integrated theoretical framework of TEs and a preliminary interdisciplinary operational definition of TEs. This examination, in turn, can help organize current research and theories, thus providing suggestions for operationalizing TEs as well as encouraging new interdisciplinary research endeavors.

## All the Facets of Transformative Experiences: Toward an Integrated Picture

As early as 1622, an example of a memorable transformation can be identified, in the work of renowned sculptor Bernini, in his representation of the precise moment in which Daphne, while fleeing from Apollo, physically transformed herself into a laurel tree. In Ovid’s Metamorphoses (Fantham, 2004), which inspired Bernini, transformation was conceived as a sudden and unexpected phenomenon, which irreversibly changes the state of things. More recently, in 1999, the Wachowskis staged one of the most famous transformative moments in the history of cinema, within their masterpiece “The Matrix.” Red or blue pill? The main character, Neo, faced the choice of whether to continue living in his habitual illusory world, or to discover the true reality, thus, embarking upon a change with no way back.

Although these fascinating examples of transformation may suggest that this phenomenon could pertain more to the domain of art and fiction than to that of reality, evidence has shown that transformation – at least, several instances of it – may occur in any moment of an individual’s life (e.g., Pearsall, 2007). The current COVID-19 pandemic could be taken as an example, in terms of events and consequences deriving from it that may be considered *transformative*. For instance, suddenly and unexpectedly, the pandemic has prompted people to change their daily routine as well as their personal view of the world, themselves, and of others (Marmarosh et al., 2020; Vos, 2021). It is not unusual to read about people changing their lives, their jobs, divorcing, or moving away from home.

Anecdotally, many readers could identify with the above descriptions of transformative experiences (TEs), and several definitions and types of TEs already exist; nevertheless, an interdisciplinary cross-domain operational definition of this complex phenomenon is yet lacking. However, given the consequences of personal transformation, understanding its underpinnings, its elicitors, as well as the boundaries of this process has become an urgent scientific issue.

The complexity within the scientific investigation of TEs unfolds through three levels. First, the exceptional and fascinating nature of this topic has garnered the interest of different disciplines across the years (e.g., James, 1902; Maslow, 1962; Mezirow, 1978; Turner et al., 1986; Bruner, 1991; Calhoun and Tedeschi, 1995; Miller and C’de Baca, 2001; Brown, 2009; Stone, 2014; Gaggioli, 2015; Yaden et al., 2017; Kason, 2019), but this endeavor has yet to establish an integrated operational interdisciplinary definition of the term TEs. Specifically, most researchers agree that TEs can be conceived as phenomena able to engender long-lasting, irreversible, pervasive consequences on individuals’ beliefs, perceptions, identity, and values (for an overview, see White, 1993; Brown, 2000; Paul, 2014; Gaggioli, 2016). However, this definition captures just one side of the process. Conversely, phenomenological features, elicitors or facilitating conditions enabling a transformative change are still open issues, which have been investigated separately.

Secondly, each discipline (e.g., anthropology, philosophy, psychology, neurobiology, education) has defined and investigated this construct at different levels of analysis. For instance, anthropology has defined this phenomenon at a meso-level of analysis, as strictly related to the specific experience of passage rites in which the central component would concern the break between past and future identities (Van Gennep, 1908). Philosophy has adopted a broader and a higher-level view of TEs’ analysis by focusing on their definitory characteristics (Paul, 2014; Carel and Kidd, 2020). Psychology has addressed mainly the micro-level of analysis, by elucidating elicitors, correlates, and effects, which have been also used to categorize and distinguish various types of TEs. Finally, a recent neurobiological model by Brouwer and Carhart-Harris (2020) suggested specific neurological correlates and mechanisms. Education has adopted a separate view on TEs, mainly relying on transformative learning theory (Mezirow, 1997, 2000), which outlines the steps to make core frames of references malleable to change. Finally, a recent neurobiological model by Brouwer and Carhart-Harris (2020) introduced the construct of “pivotal mental states” (PiMSs), defined as “transient, intense hyper-plastic mind and brain states” (p. 320), to indicate unique states mediating psychological transformation. While the perspectives of various disciplines can contribute to the richness of the understanding of TEs, a synthesis of diverse insights can facilitate research efforts and results.

Different disciplines have focused more on some instances of TEs, instead of others. For instance, in clinical psychology, an increasing attention has been devoted toward traumatic experiences and, recently, to post-traumatic and post-ecstatic experiences (Calhoun and Tedeschi, 2006; Roepke, 2013). In experimental psychology the focus has been placed on the concept of complex emotions (Gaggioli, 2015; Chirico et al., 2016). In anthropology, there is a long tradition in the study of rites of passage (Van Gennep, 1908). In the field of education, the focus has been on the process of transformative learning as achieved by means of disorienting dilemmas (Mezirow, 1997, 2000).

This has brought forth “varieties of transformative experience,” which still need to be captured within a comprehensive picture including efforts to elucidate their underpinnings. Moreover, some efforts have focused only on specific types of TEs. For instance, White (1993) and Brown (2000) introduced a model on “exceptional human experiences” including near-death experiences (NDEs), encounter-type experiences, and out-of-body experiences (OBEs) analyzed at the psychological and phenomenological level.

In this conceptual analysis, we built upon these theoretical contributions, which were integrated with a more grounded approach, focusing on specific interdisciplinary and cross-domain types of TEs. By elucidating wide varieties of TE, we moved toward an integrated picture of these phenomena, involving a preliminary interdisciplinary operational definition of TEs, using the following methodology. First, all models, theories, and empirical evidence on TEs across specific disciplines (anthropology, philosophy, psychology, neurobiology, and education) and domains (spiritual, religious, technological, educational) were analyzed and integrated with specific instances of TEs that cannot be framed within a specific discipline or domain. Then, the analysis focused on distinctiveness and commonalities among these types of TEs. This turned into indications for achieving a preliminary integrated operational definition of the varieties of TE. Finally, new research directions to improve our understanding of transformative change were presented and discussed.

### A Theoretical Overview

The need for the scientific study of transformation can be traced back to basic evidence. Some types of change can appear as different from others because they occur suddenly, unexpectedly, and without clear clues (Hayes et al., 2007; Paul, 2014). Crucially, these types of changes can deeply impact an individual’s life in an unpredictable manner (Hayes et al., 2007); they also can occur in different cultures (Carel and Kidd, 2020), stages of life (Mezirow, 1997), and in response to different (potential) elicitors (Gaggioli, 2016) or apparently spontaneously (Hood, 2014).

Given the multifaceted, universal, and impactful nature of these phenomena, it is not surprising that several disciplines have sought to understand their essence, describing their functioning, their elicitors, and how to reproduce them. Here, first, we focus on specific disciplines that provide well-established accounts of TEs. Then, examples of acknowledged interdisciplinary instances of TEs are presented and examined.

#### Anthropology

An anthropological account of transformative change provided in Van Gennep’s theory “rites of passage,” can evidence the intrinsically paradoxical or ambiguous nature of TEs elicitors. Specifically, these unusual circumstances would be able to trigger a *liminality* space (Van Gennep, 1908) – “a transformative middle-space in which individuals find themselves in between past and future identities” (Gaggioli, 2015, p. 115) – enabling transformation. Turner expanded the concept of liminality by introducing the concept of *liminoid spaces* (Turner, 1974) – out-of-the-ordinary experiences – that can be found in leisure, arts, and sports, aside from productive labor. Crucially, these are moments of freedom in which a “ludic recombination” (Turner, 1974, p. 61) of cultural factors occurs. Contemporary instances of liminoid spaces could be exceptional experiences far from the ordinary routine, which are highly memorable, very special, emotionally charged, and potentially life altering (Jefferies and Lepp, 2012, p. 38). For example, these could be unusual journeys (e.g., pilgrimages) (Kirillova et al., 2017) or extreme sports (e.g., white water rafting, spelunking, or base jumping) (Arnould and Price, 1993; Gaggioli, 2015). According to this perspective, then, there should be an out-of-ordinary elicitor/facilitating condition, acting as a liminoid space, to enable transformation. These moments would create a unique space as a potential for recombining existing cultural norms and factors into new patterns.

#### Philosophy

According to Paul (2014), out-of-ordinary elicitors should involve the choice to profoundly live a new experience, able to change our life in important ways. However, a TE entails opacity regarding the effects of being involved in such experiences. We do not know what it will be like since we dwell in a mid-suspended moment and we “only learn what we need to know after we’ve done it, and we change ourselves in the process of doing it” (Paul, 2014, p. 4). Specifically, the seminal model proposed by Paul (2014) suggested that each TE would encompass both an *epistemic* and a *personal* dimension. At the epistemic level, a TE would allow individuals grasping forms of knowledge unreachable before (“only learn what we need to know after we’ve done it”; Paul, 2014, p. 4). At the personal level, a TE can deeply change people’ values, priorities, and self-conception deeply, thus transforming an individuals’ identity (“we change ourselves in the process of doing it”; Paul, 2014, p. 4). This personal dimension consists of an irreversible cognitive shift leading to new frameworks of reference for differently viewing ourselves and others, thus, marking a clear “before” and “after.”

Recently, Carel and Kidd ‘s (2020) work broadened Paul’s conception on the role of human agency in TEs’ emergence, by elaborating more on personal and contextual constraints and affordances that can limit human control over choices. The two scholars framed Paul’s TEs within the category of (1) *voluntary* TEs, adding two more types of TEs imposed by life: (2) *non-voluntary* (e.g., being arrested and sent to Nazi concentration camps, as in the case of Primo Levi), and (3) *involuntary TEs* (e.g., saving a child who was being hit by a car remaining severely injured).

This dimension – namely, *intentionality* – allowed for a more detailed nature of TEs’ elicitors. These inductors entail a dimension of *contingency* (i.e., the occurrence of casual and unpredicted situational conditions); *vulnerability* (i.e., the helpless and unavoidable exposure to many kinds of affliction); and *subjection* (i.e., the condition of undergoing a certain event with a lack of control over it). Moreover, this broader view on TEs included also negative and ambivalent forms of transformation. Finally, and importantly, according to this model, TEs cannot be seen just as sudden life-changing moments, instead, also as the apical result of a sequence of cumulative small ordinary changes.

#### Psychology

Contrary to Carel and Kidd’s (2020) work – which also endorsed a gradual path to TEs – in the clinical psychological domain, Miller and C’de Baca (2001), drawing from the lexicon and models of quantum physics, preferred the label “quantum change,” focusing more on the impacting nature of TEs. Specifically, a quantum change would consist of a breaking point in which a radical change must occur irreversibly. According to the two scholars, there would be two types of quantum changes, as two instances of TEs: (1) *insightful* quantum changes, that are mainly *cognitive* in nature and can be associated with insight; (2) *mystical* quantum changes, having a more *emotional* character and similar to mystical experiences (MEs) (Miller, 2004). These categorizations suggested the need for emphasizing the cognitive side over the emotional one or vice versa, according to the type of TE.

Building upon previous psychological contributions on TEs, White (2004) outlined some key psychological features of transformational change. TEs were defined as unexpected, brief experiences, usually remembered vividly, entailing enduring, and comprehensive effects (i.e., they represent a “revolution in character”; White, 2004, p. 465), in which the person acts more as a “recipient,” rather than an “initiator” (p. 464), and which are positive in nature. However, this last feature has emerged as the most questionable.

For instance, the impacting - whether cognitive or emotional - nature of TEs was further elaborated by Calhoun and Tedeschi (2006) who focused on stressful and traumatic events as key elicitors of both negative (traumatic) and positive (“posttraumatic growth,” PTG) changes that have “quality of transformation” (p. 2). (Calhoun and Tedeschi, 1995, 2006). Specifically, traumatic events can result in deep, sometimes irreversible, and negative change, in which suffering disrupts individuals’ functioning. Moreover, traumas can impact individuals’ schemas and beliefs, leading also to structured syndromes such as the posttraumatic stress disorder (PTSD). Crucially, the appraisal of these events, in terms of controllability, expectancy, and probability plays a key role in the process of coping with it (Kira, 2001). After the occurrence of those traumatic events, sense of time distortion and bodily distortion are considered precursors of posttraumatic disorder in specific circumstances (McNally, 2003).

Instead, PTG occurs when the individual, after facing a traumatic struggle, changes permanently and positively, going above and beyond resilience and finding durable benefits (Carver, 1998). PTG is defined as a “positive change experienced as a result of the struggle with trauma” (Calhoun and Tedeschi, 1995; Kilmer, 2006). This definition emphasizes the transformative quality of responding to highly stressful and/or traumatic events (Calhoun and Tedeschi, 2006). The intense and dramatic experience of trauma, indeed, fosters a powerful potential for psychological transformation, as it alters the normal stable structure of the mind (Grof, 2000). A person’s pretrauma beliefs concerning the world as a just, benevolent, and controllable place can be replaced by new views, in which the negative and the positive effects of a traumatic event are combined, thus turning into a more elaborated, and complex conception of themselves and of the world (Park, 2004). In this sense, adverse events, or potentially traumatic events (e.g., developmental adversity, disability, and mental health problems) have been indicated as diversifying experiences – highly unusual and not predictable but being able to push individuals “outside the realm of normality” (Ritter et al., 2012) – able to promote forms of creative adaptation in terms of reframing an experience using new cognitive and emotional lenses (Orkibi and Ram-Vlasov, 2019). In the end, it should be noted that not all traumas and PTGs are transformative: when they lead individuals to a relevant and permanent psychological transformation, they could be considered as TEs. Research on traumatic and stressful events has generally focused on individuals’ transformation related to suffering and turmoil events (Kesimci et al., 2005; Kashdan and Kane, 2011), thus excluding perceived positive events as potential triggers of transformative growth.

Conversely, the framework of post-ecstatic growth (PEG) (Roepke, 2013) considers life events that enhance positive emotions, such as elevation and awe, as new possible triggers able to boost personal growth (Keltner and Haidt, 2003; Fredrickson, 2004; Taubman-Ben-Ari et al., 2012). Indeed, PEG concerns the idea of thriving also after highly impacting and positive experiences, giving as a result moral growth as well as deeper and closer relationships (Mangelsdorf and Eid, 2015).

Thus, in terms of factual post-event growth, there is a significant overlap in the perceived benefits of PTG and PEG, even though they are opposite in terms of valence and triggers (Roepke, 2013). However, for the purposes of this analysis, the valence of eliciting factors and outcomes is crucial to distinguish among these experiences, which consist mainly of the same processes. More specifically, triggers can have different valence and outcomes: (1) negative valence and outcomes for traumatic events that individuals were not able to accommodate and to cope with; (2) positive valence and outcomes for PEGs; (3) a negative valence but a positive outcome for PTGs, as in this case, individuals who lived a trauma were able to accommodate and to cope with it, gaining a positive as well as transformative outcome.

#### Human–Computer Interaction

Recently, the interest toward transformative and self-transcendent experiences (STEs) has grown also in the field of human computer interaction (Gaggioli, 2016; Kitson et al., 2019), and experience design (Blythe and Buie, 2021). Specifically, Gaggioli (2016) collected all these endeavors and developed a novel framework concerning how interactive technologies [e.g., virtual reality (VR)] can be used to elicit TEs. This is the transformative experience design (TED) model (Gaggioli, 2016). Transformative features associated to effective elicitors of TEs were identified on the basis of three technological assets: (1) medium, (2) content, and (3) purpose.

Regarding the medium, immersive VR was suggested as the best candidate to invite these *technologically mediated* TEs by enhancing the ecological validity of even complex experiences in the lab, thanks to the sense of presence (Barfield and Weghorst, 1993; Riva et al., 2004, 2011). Specifically, VR can be (and it has already resulted as) a valid source for experiencing new worlds, for challenging and restructuring individuals’ cognitive schemas, and as key mechanisms and correlates of TEs (Gaggioli, 2016; Riva et al., 2016; Chirico., in press). VR, indeed, allows individuals to alter their own bodily self-consciousness, providing the illusion of being placed in a different body. This is a useful asset for generating a sense of detachment from the actual body, as in the case of OBEs, later discussed. Further, VR can also cause modulation and recalibration of time perception (Bansal et al., 2019), which is a common feature of TEs.

Crucially, drawing from Paul (2014) philosophical model on TEs, the TED model suggested that technology-based TE content should involve both epistemic and emotional affordances (Gaggioli, 2016). The former is included within circumstances designed to stimulate reflection and trigger insight (Gaggioli, 2015), as the artificial representation of disorienting dilemmas, recalling Mezirow (2000) pedagogical theory. The latter are defined as perceptual stimuli aimed to boost a deep emotional engagement by evoking feelings of interest, curiosity, inspiration, or awe.

Finally, the general purpose of a technologically mediated TE is the creation of new transformative possibilities. In this regard, such TEs should let individuals enter a space of liminality, as suggested by anthropology perspective, described, according to the definition of Turner (1974) as an unfamiliar and disorienting place that creates room for reviewing and deconstructing life meaning (Turner, 1985). In other words, allowing people to experience impossible internal and external realities through VR (i.e., by modifying their own body perception, or by letting them experience the body and the perspective of another person, or again by allowing them to explore impossible and unknown environments where physics’ laws are violated) can lead them to destabilize their own mental schemes, deconstructing and reconstructing them in a new way. To date, this approach has been effective in eliciting specific transformative emotional states (Chirico et al., 2018a), as well as key micro-mechanisms related to the emergence of a TE, such as schema violations (Ritter et al., 2012).

However, further technological media can also be deemed as possible means to elicit TEs (Gaylinn, 2005). A more traditional medium, like movies, can be considered as a catalyst for personal and social growth and development (Morin, 2005; Kaplan, 2013). Transformative potential of movies has been widely explored in the education domain, by focusing on impactful masterpieces, such as Dead Poets Society^1^ (Spirou, 2016) or Billy Elliot^2^ (Schwarz-Franco, 2016). Finally, recently, also artificial intelligence (AI) has been proposed as a powerful technology able to trigger a societal change from a narrower sectorial level to a higher radical one, and always in an irreversible way (Gruetzemacher and Whittlestone, 2022). For instance, at the narrower level, the artistic domain has been increasingly influenced by different AI applications, including more collaborative forms of artistic production in music (McCormack et al., 2020), in figurative art (Schröter, 2019), and in fashion (Kautish and Khare, 2022).

#### Neurobiology

Brouwer and Carhart-Harris (2020) provided a neurobiological explanation for the emergence of PTG or traumatic potential consequences of transformation, by relying on the theory of dynamical systems (Kielhöfer, 2011). Specifically, they suggested that when an individual is involved in a long-lasting period of crisis (chronic stress), acute stress can trigger PiMSs – i.e., “transient, intense hyper-plastic mind and brain states” (Brouwer and Carhart-Harris, 2020, p. 320) – able to mediate psychological transformation. Crucially, also the surroundings and the relational context were indicated as key factors interacting with the neurobiological system to define the quality and outcomes of a PiMS.

Pivotal mental states involve the serotonin system and its 2A receptor subtype (5-HT2AR). Various acute stressors consistently induce serotonin release, upregulating 5-HT2AR expression specifically in the cerebral cortex (Anju et al., 2010; Benekareddy et al., 2011; Godar et al., 2019). Further, direct stimulation of the 5-HT2AR can induce enhanced associative learning and psychological transformation, and it can be stimulated by both spontaneous extreme stress and relevant doses of psychedelic drugs (Joëls et al., 2006; Hefferon et al., 2009; Briere et al., 2015). The mechanisms underlying PiMSs would aid rapid and deep learning in situations of existential crisis, catalyzing psychological change when circumstances demand this. Thus, according to this perspective, *learning* – rapid and associative – was considered as an essential consequence of transformation, both stemming from spontaneous acute stress (as in traumatic and PTG experiences) or induced, for instance, by psychedelics.

#### Education

A full, comprehensive model of how transformation works and can be facilitated in the learning process can be found in the domain of education. Mezirow (1978) elaborated on the theory of transformative learning as a specific transformative process taking place in education and resulting in both a deep change in a frame of reference (Mezirow, 1997, 2000; Sawatsky et al., 2018) and a shift in thinking, which irreversibly alters the way individuals are and act (Coghlan and Gooch, 2011; Stone, 2014). The main elicitor of a transformative learning process can be a “disorienting dilemma” (Mezirow, 1997) (e.g., a field trip) (Herbers and Mullins Nelson, 2009) that challenges usual mental schemas. The disorienting dilemma would lead to a cognitive self-examination, along with feelings of guilt or shame. This leads to recognizing discontentment, thanks to which individuals can express and negotiate their change through discourse, moving to explore new options for roles, relationships, and actions. According to this view, emotions and feelings would provide not only the trigger to reflect critically, but also the substance on which to reflect deeply, so that the cognitive and emotional components become strictly related. Expected outcomes entail learners become “more inclusive, discriminating, open, emotionally capable of change, and reflective so that they may generate beliefs and opinions that will prove truer or justified to guide action” (Mezirow, 2000, p. 7). Main interdisciplinarity reported outcomes concern the following typologies: worldview; self; epistemology; ontology; behavior; capacity (Hoggan, 2016).

#### Grounding Transformative Experiences: Specific Instances of Transformation

Since not all studies on TEs can be framed within a specific discipline, we also chose a more grounded approach to capture all the studies conducted on this phenomenon, that is, we focused on instances of TEs occurring in specific domains and acknowledged as TEs. For instance, interdisciplinary works, combining psychological, neurobiological, sociological, and philosophical approaches, focusing only on a peculiar type of TE are discussed.

##### Religious Conversion

Within the religious domain, the transformation par excellence coincides with the phenomenon of religious conversion. Several sociological and psychological endeavors have successfully accounted for their emergence, features, underlying mechanisms, timeframes, role of the individual, and effects, but several open questions still remain (Snook et al., 2019). At a micro-level of analysis conversion is conceived as a core identity change (beliefs and personality). For instance, recently, it has been shown that religious conversion phenomena entail personality changes as a key effect (Stronge et al., 2021). In psychology, religious conversion has been investigated using the first-hand experiences of Tolstoy, Bunyan, Edwards (etc.). James (1902), in the *The Varieties of Religious Experience* identified two types of conversions. The *volitional type* concerned situations in which “the regenerative change is usually gradual, and consists in the building up, piece by piece, of a new set of moral and spiritual habits” (James, 1902, p. 189). The *self-surrender type*, emerged as an unconscious and involuntary surrender of the individual, after an intense internal struggle between one’s aspirations and an internal hindrance. A paradigmatic example concerns the transformation of Saul (the persecutor) into Paul, the saint, as a *Road to Damascus Moment*, which stemmed from a supernatural experience of *calling* (Yaden and Newberg, 2015). Crucially, as in Carel and Kidd’s philosophical model, also in William James’ vision, it would be possible to identify a more gradual view of TE emergence. Anthropological accounts of religious conversion have acted as a key trait d’union among different disciplines, including psychology (Rotman, 2021).

Finally, mainly the sociological perspective, at a macro-level, focused on how cultural and social factors (e.g., economy, socioeconomic status, ethnicity, etc.) can influence the identities and beliefs of a potential convert (Snook et al., 2019). Generally, current accounts of conversion suggest an active role for the convert and a low impact of external, supernatural, and irresistible forces (Snook et al., 2019).

##### Self-Transcendent, Emotionally Complex, Peak, and Mystical Experiences

Spirituality is a wide domain of study, which goes beyond the realm of religion, even though the two overlap to some extent. Within this intersection, it would be possible to include STEs, which have a strong spiritual character and can be detached from any religious tradition. These experiences can be defined as transient mental states marked by decreased self-salience and increased feelings of connectedness (Yaden et al., 2017), although as for TEs, the definition of self-transcendence varies across disciplines (Kitson et al., 2020). When a STE is highly intense, it also shows a high transformative potential (Yaden et al., 2016a,^2017^). Potential conditions facilitating the emergence of these states can be paradoxical VR environments (Kitson and Riecke, 2018), psychedelic substances (Garcia-Romeu et al., 2015), spiritual instructions, dance, prayer (Garcia-Romeu et al., 2015), meditation (Hanley et al., 2020). Also peculiar social events, such as festival and parades, designed *ad hoc*, could bring forth self-transcendent shared experiences (Neuhofer et al., 2020). Therefore, STEs do not always occur as private moments, instead, the social sharing of this experience, here, emerges as a key trigger (and not just effect or correlate) for these phenomena. At the physiological level, lower respiration rate has been found to be positively correlated to a higher level of mindfulness (Ahani et al., 2014), while on the contrary, STEs have been found to induce an increase in alpha and theta EEG power (Cahn and Polich, 2006).

Self-transcendent experiences could be placed on a continuum, as they encompass a collection of phenomena ranging from mindfulness, flow, and ST emotions to TEs of awe, peak experiences, and MEs (Yaden et al., 2017).

First, as core transformative elements of a STE, it would be possible to identify special emotional states deemed as complex and transformative (Chirico and Gaggioli, 2021b; Chirico et al., 2021c), such as the emotion of awe (Chirico and Yaden, 2018c; Clewis et al., 2022). These specific emotions encompass the two main dimensions of STE, that is, connectedness and the small self. Moreover, they can be elicited by a variety of inductors, including specific VR simulations (Chirico et al., 2021a). At the physiological level, for example, awe results as a mixed valenced emotional state, as captured by electromyographic measures (EMGs) and testified by a concurrent activation of the parasympathetic nervous system and withdrawal of the sympathetic one (Chirico et al., 2017). Positive awe has been positively related to the central alpha and the beta band. It also showed negative correlations with the gamma band (Hu et al., 2017). After an experience of awe, indeed, individuals have shown more prosocial attitudes and behaviors toward other people and nature, as well as decreased aggressivity, increased generosity (Piff et al., 2015; Stellar et al., 2018), enhanced creative thinking abilities (Chirico et al., 2018b), and decreases in the cognitive emotion regulation strategy of rumination (Lopes et al., 2020). At the same time, when the awe experience is negatively connotated, it has been associated with the feeling of fear and powerlessness, loss of self-control, uncertainty, and lowered sense of situational control (Piff et al., 2015; Stellar et al., 2017).

Secondly, peak experiences can be seen as prototypical transformative examples of STEs (Maslow, 1964) consisting in a moment of elevated inspiration and enhanced well-being that can permanently influence individuals’ attitudes toward life and that can occur in all cultures (Maslow, 1962). Several characteristics are associated with peak experiences, including the perception that the world is good, beautiful, and desirable. Additional features are mainly emotional, such as feelings of being lucky, fortunate, or graced. Other features are more related to space and time dimensions, describing usual disorientation and strain for both. Peak experiences are also known for their short duration, although time perception could be expanded. They can be observed during a learning process (Lanier et al., 1996), during peak performance, sports activities (Privette, 1983), and within the musical domain (Gabrielsson et al., 2016). They can be triggered in various contexts, also in response to psychological turmoil (Taylor, 2012), and nature exposure (Naor and Mayseless, 2020). Among frequent aftereffects, there are heightened feelings of happiness, joy, and ecstasy, as well as fulfillment, peak performance; and, generally, psychological effects are seen as dependent on the context of emergence of peak experiences (Lanier et al., 1996; Solberg and Dibben, 2019).

Finally, MEs can also occur during structured spiritual or religious practices or even unintentionally (Barrett and Griffiths, 2017). These phenomena have been deemed as a particularly intense variety of self-transcendent TEs (Yaden et al., 2017) that hinge on a sense of reality far from ordinary experiences and that are characterized by feelings of unity with the whole reality (Cardeña et al., 2017). Initial characterizations of MEs suggested – within the variety of MEs – either a mysticism of introspection or of unifying vision (Otto, 1932). Indeed, MEs alter some key aspects of consciousness, such as the sense of time and space (Hood, 1975; James, 1902; Newberg and d’Aquili, 2008; MacLean et al., 2012). In addition, if they are induced by psilocybin, they also encompass the dimensions of sacredness and positive moods (MacLean et al., 2012). For instance, Stace (1960) stated that an ultimate unity “is the very essence of all mystical experiences” (p. 132), which can be further detailed as introvertive (characterized by a pure consciousness overcoming the boundaries of space and time) vs. extrovertive (featuring an “inner subjectivity of life in all things”; Stace, 1960, p. 131). These two types of MEs also share a sense of objectivity, peace, sense of sacredness, paradoxicality, and supposed ineffability (Wulff, 2014). At the emotional level, MEs entail mixed feelings ranging from fear to intense positive feelings (van der Tempel and Moodley, 2020). At the phenomenological level, these experiences have been described as brief, ineffable, and overwhelming (Yaden et al., 2017). James (1902) particularly highlighted that MEs possess *ineffability* and *noetic* quality (i.e., they reveal an otherwise hidden or inaccessible knowledge), and, sometimes, a sense of passivity and transiency. Other authors stated that MEs entail the perception that the self is perfectly integrating with one’s surroundings (James, 1902; Stace, 1960; Newberg and d’Aquili, 2000; Hood, 2002). Overall, MEs are associated to positive psychological outcomes, such as an enhanced sense of connectedness, meaning in life, positive affect (e.g., more compassion toward self and the others), or a deeper sense of identity (Brett, 2010; Nixon, 2012; Garcia-Romeu et al., 2015; Chirico et al., 2022).

When MEs are spontaneous they can maximally challenge an individual’s worldview, sometimes triggering emotional distress, confusion, and increased severity of previous psychological problems (for an overview, see van der Tempel and Moodley, 2020). When MEs are induced, they tend to reinforce previous religious schemas (Pargament et al., 2005).

Crucially, MEs can emerge in different ways, such as during sacred ritual, aesthetic experiences, physical illness, and meditation. Recently, Evans and Lynn (2021), showed that even a brief 5-min hypnosis induction could foster MEs in the lab. Longer hypnotic inductions can result in high levels of MEs in the lab (from a moderate to a great degree) in one-third of participants, as a function of their hypnotic suggestibility. MEs can be occasioned also by the psychedelic substance psilocybin in laboratory settings (for an overview, see Johnson et al., 2019), resulting in experiences comparable to those occurring in other settings both deliberately facilitated and occurring apparently spontaneously (Hood, 2014; Yaden et al., 2017b). At the neural level, the disintegration of different brain networks has been associated to a sense of dissolved self and altered salience processing (Carhart-Harris et al., 2016; Wahbeh et al., 2018).

##### Exceptional Bodily Experiences

The role of the body represents a crucial aspect that has not emerged before from the analysis of TEs provided within each discipline. Specifically, there is a class of TEs in which the body acquires a central role. It is the case of exceptional bodily experiences, in which the transformation is canalized and expressed through the body. This class of TEs can include: (1) kundalini awakenings; (2) NDEs; (3) OBEs.

In Hinduism, *Kundalini awakenings* reflect the release of inner energies through various forms, accompanied by several specific physical phenomena, such as feelings of energy in the hands, deep ecstatic sensations, and awareness of energy currents flowing through the body (Taylor, 2015; Woollacott et al., 2020) from the base of the spine (Greyson, 1993). These physical sensations are associated with a sense of joy and deep interconnectedness with others (Grey, 1985; Ring and Agar, 1986; Ossoff, 1993; Kason and Degler, 1996; De Lubac, 1999; Kason, 2019). The energetic awakening involves feelings of expansion and a dissociative and conscious awareness of leaving the body, and a sense of being enveloped in light or love (Woollacott et al., 2020). It can range from mild to intense feelings of joy to dramatic states (Taylor, 2017). As a result, kundalini awakenings entail long-term transformations in *beliefs* and *values*, including an increase in love for family, the desire to serve others, a belief in spiritual immortality, as well as deeper spiritual insights (Woollacott et al., 2020). According to western researchers, kundalini awakenings can occur in several circumstances, either spontaneously or induced (e.g., meditation, psychological turmoil, psychedelics), and they show a certain degree of overlap with NDEs (Ring, 1984; Greyson, 1993, 2000; Sanches and Daniels, 2008; Taylor, 2012).

In NDEs, – an acronym coined by Dr. Raymond Moody (1975) – the body is a key element (Holden et al., 2009). This non-ordinary state of consciousness emerges in response to a real or perceived proximity to death and entails – among other phenomenological correlates (Greyson, 1983) – the perception of leaving the body boundaries, traveling through a tunnel, and of being in front of an irreversible threshold (Martial et al., 2020). Individuals who survive them share a transformative sense of cosmic unity, transcendence of time and space, deep positive mood, sense of sacredness, noetic quality of intuitive illumination, paradoxicality, ineffability, transiency, and persistent positive aftereffects (Noyes and Slymen, 1979; Greyson, 2006). NDEs’ transformations are deep and long-lasting, with permanent and usually positive results (Morse, 1994; Simpson, 2001). For a comprehensive overview of NDEs, see Greyson (2015).

Although NDEs share many similarities with OBEs – and actually, may include OBEs (Blanke and Arzy, 2005; Blanke et al., 2016), they are quite different. Their major point of difference is that OBEs, conversely to NDEs, are not necessarily perceived as a threat to one’s life, conversely to NDEs (Nelson et al., 2006). OBEs are states during which the self appears to occupy a position spatially apart from the experiencer’s body (elevated extracorporeal location) (Blanke et al., 2016), involving both visual and somesthetic perception in which one’s own physical body is illusory (Smith and Messier, 2014). OBEs’ transformative impact on an individual’s psychological well-being has been recognized (Riva et al., 2016), followed by an increasing interest in their healing potential (Sellers, 2019). OBEs and NDEs are similar both for their transformative outcomes and their nature, as they involve a dissociation from the body and hyper-real sensorial, perceptual, cognitive as well as affective processes (Blanke et al., 2016). NDEs are even less common than OBEs, with OBEs generally occurring rarely in lifetime (no more than once or twice) (Blanke et al., 2016).

Kundalini awakenings, NDEs, and OBEs can be considered three different manifestations of a sudden transformation unfolding through the body. However, the elicitors (or triggers) most frequently associated to these kinds of TEs are different. For example, elicitors could be traumatic events for NDEs (Greyson, 2000), e.g., cardiac arrest (Van Lommel et al., 2001), and meditation for kundalini awakenings (Woollacott et al., 2020). Crucially, OBEs can be caused, besides specific pathological conditions (e.g., depression, personality disorders), pharmacological substance consumption (e.g., LSD, marijuana, etc.), stimulation of specific brain areas (i.e., the temporoparietal junction, TPJ; Bos et al., 2016) or general anesthesia, also by the simulation of multisensory conflicts between a visual stimulus and a tactile, vestibular, or cardiac stimulus induced in a VR setting, by means of videos or robotic devices (Blanke, 2012; Fernandez-Alvarez et al., 2021).

##### Psychedelically Induced Experiences or Psychedelic-Like Experiences

Increased attention has been given to psychedelics-induced experiences (Fadiman and Kornfeld, 2013), specifically, with regard to peculiar chemical triggers of TEs: psilocybin administration provided one of the first models for experimentally controlled investigation of quantum changes (Barrett and Griffiths, 2017). Under supportive conditions, psilocybin can foster deeply meaningful and significant experiences (e.g., mystical-type experiences, which are discussed later in detail) (Pahnke, 1963; Griffiths et al., 2006, 2011). Follow-up studies at 2 and 14 months confirmed that psilocybin experiences enabled participants to change durably and positively their attitudes and behaviors, and consistently with what community observers stated (Griffiths et al., 2006, 2008). Attributions to the psilocybin experience included changes in attitudes, mood, altruism, and other behaviors, as well as interpersonal closeness, gratitude, life meaning/purpose, forgiveness, death transcendence, daily spiritual experiences, religious faith, and coping (Griffiths et al., 2018).

Ibogaine was also found to have similar transformative effects too (Brown et al., 2019). Observational studies have described participants’ increased reflection, forgiveness, and self-forgiveness (Brown et al., 2019), which allowed them to enhance empathy and to attain relief from guilt (Heink et al., 2017), thus enabling personal transformation. Also quantitative results (Brown et al., 2019) sustained ibogaine’s psychotropic effects as being psychologically profound, leading to far reaching transformations (pp. 3–4), which is consistent with numerous other studies exploring ibogaine (Naranjo, 1969, 1974; Lotsof and Alexander, 2001; Schenberg et al., 2014; Heink et al., 2017; Camlin et al., 2018; Rodger, 2018) and other hallucinogens’ effects, such as DMT, LSD, as well as mescaline (Hood, 1975; Griffiths et al., 2008; MacLean et al., 2011). For an up-to-date review of long-term effects associated to psychedelic drugs consumption, see Aday et al. (2020).

Crucially, it has been showed that it was possible to experience drug-like effects without consuming a drug but just by being close to someone who did so (Tart, 1971). Indeed, the effects of psychedelics originated also from contextual factors, including previous expectations. In a recent single-blind between-subjects study (Olson et al., 2020), some participants, belonging to the placebo group, reported intense alterations in consciousness, which had been usually found associated with moderate or high doses of psychedelics.

These controlled drug experiences, similar to mystical ones, incorporated the dimensions of *unity*, sacredness, positive mood, transcendence of time/space, ineffability (MacLean et al., 2012), and of being overwhelming both cognitively and emotionally. Like MEs, these states could be emotionally ambivalent, as they could involve not only positive emotions but also regret, fear, anxiety, and upset (Brown et al., 2019).

Specifically, at the phenomenological level, several authors have also tried to outline the unique experiential profile of psychedelic experiences (e.g., Preller and Vollenweider, 2016), and this phenomenon has been conceived as a dynamic process lying on a perception–hallucination continuum, which is characterized by an increasing arousal and by the loosing of ego boundaries (Preller and Vollenweider, 2016).

## An Interdisciplinary Integrated Picture of Transformative Experiences: What is Shared and What is Different

The initial comprehensive analysis conducted so far by giving voice to all the acknowledged interdisciplinary varieties of TE provides a foundation for a new integrated picture of these phenomena. Hereinafter, first, we focus on the redundant characteristics that frequently have emerged within different domains and disciplines’ discourse by applying the reasoning by analogy approach (Ketokivi et al., 2017). We provide an overview of the outcomes of this analysis in Table 1. Then, shared elements (at any levels, from the biological to the existential one) and distinctive features of each TE are discussed.

**TABLE 1:** Overview TEs instances’ main features: main reference discipline(s), domain(s), phenomenological correlates, elicitors, and aftereffects.

TE instance	Main involved discipline(s)	Domain	Main reference(s)	Description	Epistemic expansion	Emotional complexity	Elicitors/ Facilitating conditions	Aftereffects
Religious conversion	Psychology, philosophy, anthropology, sociology	Religion, spirituality	James, 1902; Ullman, 2013; Snook et al., 2019	A moment of enlightenment, self-surrendering, and union with a new religious awareness of superiority consisting in a process through which persons move from their previously held religious beliefs to the beliefs of a new religious tradition.	Perception of new truths that were inaccessible before; time perceived as stopped, slowed down, or dilated; brief duration.	Sudden loss of all concerns, relief, sense of peace, harmony, deep happiness, faith. High emotional intensity.	Current theories: key active role of the convert, lower impact of external supernatural causes, more emphasis on converts’ need for meaning and purpose, despite also cultural factors play a role.	Key personality changes (e.g., increase in honesty-humility, conscientiousness, and neuroticism after the conversion); key identity changes; new language; new beliefs.
Self-transcendent and emotionally complex experiences	Psychology, neurobiology, nursing, psychiatry, design, human computer interaction	Miscellaneous	Garcia-Romeu, 2010; Yaden et al., 2016a,^2017^; Kitson et al., 2020	The *transient* mental state marked by the transcendence from the material and physical limitations, and by a deep connection with something greater than oneself.	Perception of self-diminishment and decreased self-salience; time is perceived as unbounded.	Self-transcendent positive emotions: elevation, compassion, gratitude, love, and awe. High emotional intensity.	Meditation, peculiar social events hinging on connection with others, virtual reality paradoxical scenarios, nature; catalyzed with spiritual instruction, dance, prayer, and psychedelic substances.	Decreased anxiety, increased energy, insight, social ability, and sustained positive affect, value re-orientation, increased concern for others, increased positive affect, and disidentification from old patterns of thinking or behavior; increased prosociality (toward other people and nature); increased generosity, enhanced creative thinking; decreased ruminative strategies. The negative counterpart of ST emotions emerged as associated to highly intense fear and powerlessness, loss of self-control, uncertainty, and lowed sense of situational control.
Peak experience	Psychology, neurobiology	Miscellaneous	Maslow, 1962, 1964	A *sudden* and *unexpected* acceleration of personal development or self-actualization.	The appraisal of the world as good, beautiful, desirable, sudden certainty that polarities and dichotomies have been resolved, strain in both time and space, rapid duration while perceived time is expanded.	High inspiration, awe, wonder, gratitude, deep well-being, high emotional intensity.	Exposure to nature, sport, music, spiritual and religious context, learning.	Effects depends on the context in which the peak experience takes place; individuals recognized immediately this experience as a turning point, achieving peak performance; highest positive feelings (joy, happiness, and ecstasy).
Mystical experience	Philosophy, psychology, neurobiology	Religious, spiritual	James, 1902; Stace, 1960; Newberg and d’Aquili, 2000, 2008; Hood, 2002	Particularly intense variety of self-transcendent experiences (Yaden et al., 2017). An experience that tends to occur suddenly, it is often transient, it appears as ineffable, joyful, it involves the perception of an ultimate unity, of oneness; transcendence of the ego; a full conviction of immortality; and it tends to be attributed supreme value. Some people interpret MEs as experiences of unity with God (Thalbourne, 2003).	Perception of vanishment of the whole self, cognitively overwhelming, ineffable, noetic quality, sacredness; strain in space and mostly in time while it has a short duration.	Mixed feelings raging from fear to intense positive affect.	Psychedelics; hypnosis; meditation; sacred ritual, aesthetic experiences physical illness.	Enhanced sense of connectedness, meaning in life, positive affect (e.g., more compassion toward self and the others) or a deeper sense of identity.
Kundalini awakening	Psychology, anthropology, sociology	Spirituality, self-transcendence	Taylor, 2015; Kason, 2019; Woollacott et al., 2020	Exceptional physical experience consisting of a huge release of energy, accompanied by temporary corporeal symptoms.	Conscious awareness of leaving the body, increased sensory sensitivity, deep interconnection with others; time is perceived as loosened, as the sense of linear time was lost (i.e., “out of time” experience).	Deep ecstatic sensations, joy, enhanced sense of connectedness and unity, reduced fear of death, feelings of expansion, envelopment in love or light; possible dramatic negative emotions.	Meditation, psychological turmoil, psychedelics.	Change in beliefs and values, reduced tendency to aggression; possible negative cognitive outcomes that leave an indelible mark, as disruptions of psychological functioning and mental illness; new sense of identity.
Near-death experience	Psychology, philosophy, neurobiology	Miscellaneous	Greyson, 2000, 2006, 2015; Simpson, 2001; Holden et al., 2009	Altered state of consciousness on the (real or perceived) threshold of death. Major focus on the peculiar feeling of leaving the physical body and encountering non-physical entities/ environments.	Accelerated thoughts; life review; perception of understanding everything, flash from the past, perception of leaving the body boundaries, and of traveling through a tunnel and of being in front of an irreversible threshold; absence of time and space.	Sense of cosmic unity and sacredness, peace, positive mood, feelings of harmony, unity, joy, revelation, and connectedness.	Meditation, psychological turmoil, psychedelics; alternations in oxygen levels; neurological alterations.	New responses to life-threatening dangers, life review, sense of being controlled by an outside force, transformation of attitudes, shift to a new belief system, decreased death anxiety, heightened spiritual awareness.
Out-of-body experience	Psychology, philosophy, neurobiology	Miscellaneous	De Foe et al., 2012; Smith and Messier, 2014; Sellers, 2019	States during which the self appears to occupy a position spatially apart from the experiencer’s body (elevated extracorporeal location) (Blanke et al., 2016).	Disembodiment; the self appears occupy an elevated extracorporeal location; enhanced reality, hyper-real cognitive perception, extremely vivid stimuli, and settings, intensified sensory inputs that lead to transformative outcomes.	Highly intensified emotions, hyper-real affectivity.	Pathological conditions (e.g., depression, personality disorders), pharmacological substances assumption (e.g., LSD, marijuana, etc.); stimulation of specific brain areas (temporoparietal junction area, TPJ); general anesthesia, also by the simulation of multisensory conflicts between a visual stimulus and a tactile, vestibular, or cardiac stimulus induced in virtual reality (VR), by means of videos or robotic devices.	Changes in bodily self-consciousness (self-identification and self-location); decreased fear of death; dissociation.
Trauma	Psychology, philosophy, clinical medicine, neurobiology	Clinical	Calhoun and Tedeschi, 1995, 2006; Tedeschi, 1999; Grof, 2000	Radical changes given by the experiencing of a negative high-impacting event. If changes are negative, the TE leads to trauma. If changes are positive, the TE turns into posttraumatic growth.	Expectancy, probability, and controllability evaluations associated to the events; sense of time distorted and bodily distortion as predictors of PTSD.	Terror, perception of threat.	Negatively overwhelming psychological stressors individuals could not cope with.	Altered self-capacities, mood disturbance, enhanced avoidance responses, posttraumatic stress.
Posttraumatic growth	Psychology, philosophy, clinical medicine, neurobiology	Clinical	Calhoun and Tedeschi, 1995, 2006; Tedeschi, 1999; Grof, 2000	Positive change experienced as a result of the struggle with traumatic events.	Expectancy, probability, and controllability evaluations associated to the events; sense of time can be distorted.	Terror, perception of threat; relief.	Negatively overwhelming psychological stressors individuals could cope with; the perception of the triggering event depends also on individual differences, e.g., the degree of previous religiosity.	Personal development, enhanced authenticity responsibility toward oneself and others, accepting attitude to death, increased self-confidence, new identity, values, and perspectives.
Post-ecstatic growth	Psychology	Miscellaneous	Fredrickson, 2004; Roepke, 2013; Mangelsdorf and Eid, 2015	Radical positive changes given by the experience of highly impacting positive events.	The relevance of time and space varies according to the ecstatic or peak experience, which generally involves a transcendence of these dimensions.	High positive emotional valence, which can be associable to the peak experience’s one.	Positive affective experiences, awe moments.	Durable and positive changes regarding appreciation of life, relationships, enhanced spirituality, renewed life meaning, and personal strengths.
Psychedelic experience	Psychology, psychopharmacology, neurobiology, anthropology	Miscellaneous	Barrett and Griffiths, 2017; Camlin et al., 2018; Griffiths et al., 2018; Brown et al., 2019; Brouwer and Carhart-Harris, 2020	Dynamic process lying on a perception–hallucination continuum, which is characterized by an increasing arousal and by the loosing of ego boundaries.	Space and time transcendence; ineffability; overwhelming in nature; unity, ego-dissolution; perceptual illusions (alterations of the environment and of the body image; peculiar visual phenomena); deep insights into the nature and structure of the universe.	Gratitude, forgiveness, unity, death transcendence sacredness, positive mood, but also regret, fear, anxiety, and upset.	Typically elicited by psychedelic substances (e.g., psilocybin; Ibogaine; DMT; LSD).	Positive changes in attitudes and behaviors, increased positive coping, prosociality, and empathy. Negative long-term changes at the neurological, personality, molecular, and psychological level (see Aday et al., 2020).
Transformative learning experience	Education, psychology, philosophy	Learning	Mezirow, 1978, 1997; Kleiber et al., 2002; Stone, 2014	Process of changing accustomed assumptions, thus producing an effective shift of reference frameworks.	Deep and structural shift in mental schemas, beliefs, and perspective, loss of old meaning perspectives to find new selves, heightened self-reflection.	Emotional and social learning, hope, newness, intense emotions as drivers for self-reflection, but also guilt, shame, disorientation, dissonance.	Disorienting dilemma.	Both positive and negative outcomes, e.g., changes in worldview, schema and paradigm, changes concerning how learners conceptualize themselves, and how they related to others or to the world in general; increased empowerment/responsibility; new ways of knowing, which is more open, discriminating, extrarational. Development of new skills. Implementation of new social actions, which are consistent with epistemological changes; heightened spirituality.

*Quantum change has not been included since it has been often considered as an overall experience encompassing mystical experiences and the phenomenon of insight. Liminality as well has been indicated as a broader anthropological framework for capturing transformative experiences, while PiMSs address mainly the neurobiological level of a transformative experience, thus, acting as an explanatory neurobiological model of a special phase of the transformative process.*

### Applying the Reasoning by Analogy Approach

As a first step, we focus on the shared aspects across different instances of TEs, at a higher level. To this end, we drew from a reasoning by analogy approach (Ketokivi et al., 2017). This methodological approach, which was proposed by Ketokivi et al. (2017), relies on the concept of analogy, whose value has been recognized across all the sciences. (Hesse, 1966), since it provides links between two or more different domains of knowledge (Gentner and Markman, 1997), exploiting their similarities regarding one specific phenomenon, in order to make inferences that connect them (Gentner and Namy, 1999; Ketokivi et al., 2017). Analogies have proven to be effective in enabling progress for both research and theory, especially in boundedly rational and resource-constrained contexts, since they allow researchers to focus and deepen only the relevant part of the phenomenon, abstracting out other parts, and overcoming discrepancies (March, 1994; Ketokivi et al., 2017). Here, this approach permits to combine knowledge from different domains on TEs, in order to elucidate their transversal functioning. Then, we move to consider how each TE fulfills the shared elements differently, thus bringing forth varieties of TE.

From the implementation of the reasoning by adopting analogy approach, four specific high-level shared elements emerged that can be used to frame the multifaceted nature of TEs. Firstly, these concern, first, TEs’ phenomenological dimensions (i.e., characteristics subjectively perceived by individuals during TEs). These features can be further divided into: (1) epistemic expansion (new forms of knowledge of the self and of the world) and (2) emotional complexity, involving intense, mixed emotions, and emodiversity (Berrios, 2019). In addition to the phenomenological elements, two psychological elements can be identified: (3) facilitating conditions/elicitors, and (4) specific effects on the recipient (aftereffects).

The **epistemic expansion** component concerns new forms of knowledge about the self and the world. For instance, in STEs, a sense of self-diminishment and of being connected with all beings is a predominant result. Peak experiences entail the appraisal of the world as a good, beautiful, desirable, place, and the awareness that all polarities and dichotomies have been resolved. In NDEs, there is a dissolution of body boundaries, perception of being able to suddenly understand everything, and being on the edge of an irreversible threshold. Moreover, subjective time perception is recurrently expanded or dilated, while the experience itself could last even a few moments. Space perception is also often strained, distorted, and transcended, as in the case of OBEs and NDEs. This dimension of altered time perception, instead, was less relevant (if not absent) in Mezirow’s transformative learning process. However, during transformative learning, the disorienting dilemma acted as a central moment of belief shift through the violation of previous expectancies. Finally, Brouwer and Carhart-Harris (2020) model on PiMSs could provide a particularly suitable framework to address the neurobiological underpinnings of this epistemic expansion component, which reflects the phenomenological transversal dimensions shared by all the types of TEs considered so far.

The **emotional complexity** component deals with the extremely intense and mixed nature of feelings, affects, and discrete emotions involved during ongoing TEs. Extremely intense feelings can be found during traumatic events, OBEs, NDEs, or STEs. Emotional complexity mainly refers to the experiencing of positive and negative states together (i.e., emotional dialecticism) (Larsen et al., 2001; Spencer-Rodgers et al., 2010) and to the experiencing of emotions with high granularity, with a wider variety of discrete emotions reported (i.e., emotional differentiation) (Kashdan et al., 2015). Although the literature has often evidenced generally positive emotional characteristics of TEs, a deeper overview of these experiences showed that they are characterized by mixed emotions – involving the co-activation of opposite emotions at the same time – or by emotional variability (Larsen et al., 2001; Berrios, 2019). There could be two main mechanisms in which mixed emotions underlying TEs could work to give rise to these experiences. According to the bipolar view (Russell, 2017), mixed emotions may be felt when individuals have already undergone a transformation, but they still emotionally fluctuate between rejection and acceptance of a chosen behavior. Conversely, the bivariate view by Larsen (2017) suggests that an event could be characterized by two simultaneous opposite feelings (e.g., fear and happiness at the same time) (Roseman, 2017). People living a TE are likely to experience a diverse and abundant array of emotions, ranging from ecstasy, bliss, and relief (e.g., as in religious conversions) to guilt, fear, and regret (see psychedelic-induced MEs in Brown et al., 2019). The breadth of TEs’ emotional repertoire can suggest that these experiences are characterized by *emodiversity*, which refers to the richness of emotional complexity (Quoidbach et al., 2014; Berrios, 2019).

Finally, TEs can usually be conceived as either spontaneous events or as induced by specific facilitating conditions, also by recognizable elicitors. **Facilitating conditions/elicitors** for each TE analyzed in this work are reported in Table 1.

Some specific state-conditions act as recurrent triggers of different TEs (psychedelics, meditation, spiritual practices), others as peculiar elicitors of specific instances of TEs (e.g., cardiac arrest for NDEs) (for a finer and up-to-date list of triggers, see White and Brown, 2000). In addition, recently, more stable variables (trait-like) have emerged as potential preparatory conditions for engendering specific TEs, as in the case of PTG, which is facilitated by previous religiosity (i.e., religious affiliation and strength of religious beliefs) (Taku and Cann, 2014). Crucially, novel technological devices, such as VR, have been proposed as valid tools for inviting even more complex TEs in the lab (see OBEs in Bourdin et al., 2017; van Heugten-van der Kloet et al., 2018). VR, indeed, allows manipulating (also separately) several components of cognition and emotion (Slater et al., 2010; Slater, 2018; Bolt et al., 2021) as well as overall potentially TEs (Chirico et al., 2020).

However, if basic underpinnings of TEs are not previously captured and understood, VR can turn useless. Indeed, there is always a risk of mistaking the role of this tool in promoting this class of experiences by nurturing fallacious reasoning. It should be noted that VR can easily resemble phenomenological features typical of a given TE. However, this would not guarantee that the same underlying mechanisms/underpinnings of a TE are activated during an equivalent VR experience. Therefore, a deeper comprehension of the basic mechanisms underlying each TE (starting from their facilitating conditions) is essential to reproduce it in the lab by means of VR or any other simulation tool.

Finally, although it may look redundant, it would be useful to demonstrate that all the experiences included in this conceptual analysis show **specific effects on the recipient (aftereffects)** that would be able to impact individuals’ cognition, emotions, and personality in the long run. This has always been a basic criterion used to define TEs, as mentioned at the beginning of the present work. However, even though all instances of TEs considered so far entail long-lasting, pervasive aftereffects, they also feature a high degree of variability in terms of their consequences. This, again, could support the idea that – echoing William James’s seminal work on religious experience – it would be useful and appropriate to indicate TEs as “varieties of transformative experience” with two shared core components, elicitors/facilitating conditions particular to specific types of TEs, along with a certain degree of internal differentiation.

These components were summarized in Table 1.

### Toward a Tentative Integrated Interdisciplinary Conceptualization of Transformative Experience

In line with all the evidence reviewed and analyzed so far, a new preliminary integrated definition of TEs is here advanced.

Transformative experiences can be defined as brief experiences, perceived as extraordinary and unique, entailing durable and/or irreversible outcomes, which contribute to changing individuals’ self-conception, worldviews, and view of others, as well as their own personality and identity by involving an epistemic expansion (as new forms of knowledge of the self, others, and the world) and a heightened emotional complexity (emotional variability, high intensity, mixed emotions), as the two core phenomenological features. They are usually remembered vividly.

These experiences emerge suddenly, either apparently spontaneously or they can be invited by specific elicitors/facilitating conditions encompassing both state (related to contingencies) and trait elements (related to more stable conditions of the experiencer). Elicitors, usually, are perceived as novel stimuli, able to challenge an individual’s mental schema, thus also resulting in disruption.

Some TEs can also encompass transcendental elements. Some TEs (and some phases of a TE) can be marked by peculiar neuropsychophysiological underpinnings.

Finally, it is appropriate to conclude this section by suggesting that a more active dialog among several disciplines and domains would be not only be desirable but also especially advantageous, despite the effort that this would require.

## Conclusion

Although literature on specific types of TEs is growing and bringing forth promising results, an integrated view of this phenomenon has yet to be developed. In this conceptual analysis, we aimed to provide this interdisciplinary view by marking shared phenomenological features of TEs, as well as their intrinsic particularities, to outline a preliminary interdisciplinary operational definition of TEs.

This effort could turn, as a first outcome, into a more fluid dialog among scholars from different disciplines interested in examining this phenomenon at different levels of analysis, thus resulting in a finer and more comprehensive view of the phenomenon and its underpinnings. Otherwise, the risk can be a fragmentation of TEs’ conceptualization and research.

As a second outcome, this endeavor could lead to a paradigmatic shift, as the direction of research is moving from retrospective to predictive study of TEs. For instance, nowadays, it is only possible to sense pandemic as a *potentially* TE. An open issue is to scientifically predict the extent to which this will occur, that is, to measure its transformative potential. This would be possible only after having elucidated the underpinnings of transformation at different levels, from the biological to the existential level. In this regard, the results of this analysis could inform the design of novel, integrated, and comprehensive instruments to measure the potential of an event to engender a specific type of TE in a particular person. Moreover, future studies could also consider the possibility of developing a new comprehensive and interdisciplinary instrument capturing the “minimal transformation” embedded into an experience, also building upon the conditions for the appearance of a minimal phenomenal self (e.g., Windt, 2015b; Josipovic and Miskovic, 2020; Metzinger, 2020). This could be useful, for instance, in the assessment of health promotion interventions. Other possible research lines could elucidate and systematically analyze the antecedents/facilitating conditions giving forth different types of TEs, thus enriching the examination of these class of experiences. The aim of this work is to stimulate a fluid dialog among researchers to acquire a comprehensive view of this phenomenon, despite it should be noted that this work is preliminary in nature and needs to be constantly updated by new evidence, insights, and suggestions.

## Author Contributions

AC and EG wrote the first draft. AC conceived the rational along with AG and MP. AK and BR revised the manuscript, provide relevant suggestions, and improvements. All authors contributed to the article and approved the submitted version.

## Conflict of Interest

The authors declare that the research was conducted in the absence of any commercial or financial relationships that could be construed as a potential conflict of interest.

## Publisher’s Note

All claims expressed in this article are solely those of the authors and do not necessarily represent those of their affiliated organizations, or those of the publisher, the editors and the reviewers. Any product that may be evaluated in this article, or claim that may be made by its manufacturer, is not guaranteed or endorsed by the publisher.

## Footnotes

1 Dead Poet Society, Peter Weir, 1989.

2 Billy Eliot Steven Daldry, England, 2000.
